# “Risk factors of birth asphyxia”

**DOI:** 10.1186/s13052-014-0094-2

**Published:** 2014-12-20

**Authors:** Hafiz Muhammad Aslam, Shafaq Saleem, Rafia Afzal, Umair Iqbal, Sehrish Muhammad Saleem, Muhammad Waqas Abid Shaikh, Nazish Shahid

**Affiliations:** Dow Medical College, Dow University of Health Sciences, Karachi, 74200 Pakistan; Final Year student of Dow Medical College, Dow University of Health Sciences, Karachi, 74200 Pakistan

**Keywords:** Asphyxia, Fetal distress, Neonate

## Abstract

**Background:**

Birth asphyxia is an insult to the fetus or newborn due to failure to breath or breathing poorly, leads to decrease oxygen perfusion to various organs. According to WHO, 4 million neonatal deaths occurred each year due to birth asphyxia. Our goal was to evaluate antepartum, intrapartum, and fetal risk factors of birth asphyxia.

**Methods:**

It was a Retrospective Case control study, conducted at Neonatal Intensive Care Unit of pediatric ward (I, II, III) and in Gynecology wards (I, II, III) of Civil Hospital Karachi, Dow University of Health Sciences. Study was conducted from January 2011-November 2012. Neonates diagnosed with birth asphyxia were considered as “cases” while neonates born either with normal vaginal delivery or by cesarean section having no abnormality were considered as “control”. Demographics of both the mother and neonate were noted and Questions regarding possible risk factors were asked from mother. Ethical issues were confirmed from Institutional review board of Civil Hospital Karachi, Dow University of Health Sciences. All data was entered and analyzed through SPSS 19.

**Result:**

Out of total 240 neonates, 123 were “cases” and 117 were “control”. Mean maternal age in “case” group was 24.22 ± 3.38 while maternal age of control group was 24.30 ± 4.04. Significant antepartum risk factors were maternal age of 20–25 (OR 0.30 CI 95% 0.07-1.21), booking status (OR 0.20 CI 95% 0.11-0.37), pre-eclampsia (OR 0.94 CI 95% 0.90-0.98) and primigravidity (OR 2.64 CI 95% 1.56-4.46). Significant Intrapartum risk factors were breech presentation (OR 2.96 CI 95% 1.25-7.02), home delivery (OR 16.16 CI 95% 3.74-69.75) and maternal fever (OR 10.01 CI95% 3.78-26.52). Significant Fetal risk factors were resuscitation of child (OR 23 CI 95% 31.27-1720.74), pre-term babies(OR 0.34 CI 95% 0.19-0.58), fetal distress (OR 0.01 CI 95% 0.00-0.11) and baby weight (OR 0.13 CI 95% 0.05-0.32).

**Conclusion:**

Measures should be taken to prevent neonatal mortality with great emphasis on skilled attendance at birth and appropriate care of preterm and low birth weight neonates.

## Background

Birth asphyxia, although the correct definition is imprecise, is an insult to the fetus or newborn due to failure to breath or breathing poorly leading to decrease oxygen perfusion to various organs. According to WHO, 4 millions deaths yearly occurred due to birth asphyxia, representing 38% of all deaths of children under 5 years of age. In low-income countries 23% of all neonatal deaths occurred due to birth asphyxia [1]. According to a survey conducted by WHO in 2005, it is also one of the leading causes of neonatal deaths within first week of life [2]. It is strongly associated with 1.1 million intrapartum stillbirths and is responsible for long-term neurological disability and impairment [3]. Causes of perinatal birth asphyxia may According to WHO classification of diseases ICD10, Severe birth asphyxia is when the APGAR score at 1 min is 0–3. Mild and moderate birth asphyxia is when Apgar score at 1 min is 4-7 [4,5].

Asphyxia is a condition that occur when there is an impairment of blood-gas exchange, resulting in hypoxemia (lack of oxygen) and hypercapnia (accumulation of carbon dioxide). The combination of the decrease in oxygen supply (hypoxia) and blood supply (ischemia) results in a cascade of biochemical changes inside the body, whose events lead to neuronal cell death and brain damage. Continuous asphyxia will also lead to multiple organ systems dysfunction. Birth asphyxia is a serious clinical problem worldwide and contributes greatly to neonatal mortality and morbidity [4].

Causes of perinatal birth asphyxia may be maternal or fetal. Those who survive asphyxia at birth may have chance to develop neurological complications including epilepsy, cerebral palsy and developmental delay [6]. Risk factors of birth asphyxia has been divided into antepartum, intrapartum and fetal. Risk factors include increasing or decreasing maternal age, prolonged rupture of membranes, meconium stained fluid, multiple births, non-attendance for antenatal care, low birth weight infants, malpresentation, augmentation of labour with oxytocin, ante partum hemorrhage, severe eclampsia and pre-eclampsia, ante partum and intrapartum anemia [7,8]. The prognosis and severity of the symptoms of child with birth asphyxia depend on the risk factors and management of the patient

According to a study in Pakistan, birth asphyxia contributed to 16.52% of hospital admissions and is responsible for 21% of infant deaths [9].

### Objectives

However, Pakistan being the developing country, experiences a significant morbidity due to birth asphyxia and its sequel of complications. In Pakistan there is not much data available on the risk factors of birth asphyxia, hence studies are required to evaluate the risk factors of birth asphyxia so that interventions can be done to educate and guide people about the risk factors and management strategies. Our main goal was to evaluate the antepartum, intrapartum and fetal risk factors of birth asphyxia.

## Methodology

### Study setting

It was a Retrospective case control study conducted at the neonatal intensive care unit of pediatric ward (I, II, III) and at Gynecology ward (I, II, III) of Civil Hospital Karachi, one of the largest public sector, tertiary care hospitals in Karachi. Study was done from January 2011-November 2012.

### Study participants

Subjects were divided into cases and control. Neonates diagnosed with birth asphyxia were considered as “cases” while neonates born from normal vaginal delivery or by cesarean section with no abnormality were considered as “control”. Sample size was determined with the help of Open EPI calculator.

Diagnostic criteria of Birth Asphyxia:

- Gestational age after viability.
- Severe Asphyxia: Apgar score at 1 min is 0–3.

Mild-Moderate Asphyxia: Apgar score at 1 min is 4–7

c) Absence of major congenital malformation.
d) Multi organs failure in the first 72 hours or convulsion in the first 24 hours of life.
e) History of delayed cry.

Exclusion Criteria were:

- Birth weight less than 1000 g pre-maturely.
- Opium or Anesthesia related Low APGAR score.
- Babies with lethal anomalies like hydrops, cyanotic congenital heart defects, congenital or chromosomal anomalies and congenital infections.

### Study protocol

Apgar scoring consists of five physical sign: heart rate, respiratory effort, reflex irritability, muscle tone and color. Labor was considered prolonged when it exceed 12 hours in primigravida or 8 hours in multipara [10]. Fetal distress was diagnosed when fetal heart rate was recorded at less than 100/mm or more than 160 min or when it was irregular. Prolonged rupture was labeled when rupture of membranes was more than 18 hours before the birth of baby. Temperature of more than 99 F was considered as febrile. Anemia in antenatal period was defined as documented evidence of hemoglobin measurement of less than 11 gms/100 milliliter at any time prior to or after admission as emergency referrals. Premature rupture of membranes is defined as a condition in which rupture of the membrane of the amniotic sac and chorion occur more than one hour before the onset of labor. Information regarding diabetes, acidosis, striol level, surfactant test and ultrasound guided abnormalities were extracted from medical records.

Intrapartum: Event occurs during labor and delivery.

Antepartum: Event occurring before childbirth, with reference to the mother.

Maternal characteristic were obtained during interview of mothers, using pre-structured Performa.

## Consent

Before entry into the study, subjects were informed about the aims, methods and anticipated benefits. Subjects will be informed that their participation is voluntary. They will be informed that choosing not to participate will not affect their care. After giving sufficient information written consent was obtained and confidentiality of data was maintained.

### Questionnaire

Performa was filled on each case. Questionnaire comprises of 24 questions. 1st portion comprise of demographic data of neonate and mother. Q1-Q7 comprises of questions regarding age of mother, type of case (booked or not), any illness during pregnancy, drug and smoke history, type of pregnancy, history of diabetes, anemia and any previous history of birth asphyxia. Second section comprise of history of current pregnancy. It consist of questions from Q8-Q18, these were regarding any fetal condition diagnosed during pregnancy, presentation of fetus, mode of delivery, type of assisted vaginal delivery, anesthesia received during C-section, history of prolong labor, place of delivery, any emergency complication face by mother and history of sedation. Last section was regarding neonatal characteristic. It compromise of questions from 19–24, which consist of gestational age at birth, history of cry and seizure, about resuscitation, mode of resuscitation and birth weight. Information about the date and time of birth, gender, birth weight and result of Apgar score at 1, 5 and 20 minutes has been noted in questionnaire.

### Ethical certificate

Study was approved from the institutional review board of Civil Hospital Karachi, Dow University of Health Sciences.

### Statistical analysis

All data was entered and analyzed through SPSS 19. Mean and standard deviation were used for continuous data while frequency and percentage were calculated for categorical data. Risk factors for birth asphyxia were grouped into antepartum, intrapartum, and fetal variables. Association between birth asphyxia and risk factors was determined by Binary logistic regression. The percentage of risk factors associated with birth asphyxia at different intensity levels was estimated by odds ratio with the confidence interval CI of 95%. Threshold of significance was set at 0.05

## Result

During the period of two years (January 2011-November 2012), 240 cases fulfilled the inclusion criteria. Out of these 240, 123 were of birth asphyxia (cases) and rest of 117 were normal (control). Male neonates were 147 (61.3%) and females were 93 (38.8%). Mean maternal age in asphyxia group was 24.22 ± 3.38 while mean maternal age of control group was 24.30 ± 4.04.

### Antepartum risk factors

Mothers at age of 20–25 were at higher risk of developing Birth asphyxia as compare to younger or elder mothers (<20 or >25) (OR 0.30, CI 95% 0.07-1.21,). Risk increased significantly with decline in booking status of mother (OR 0.20, CI 95% 0.11-0.37, p = <0.01). Pre-eclampsia was associated significantly with increase risk of birth asphyxia (OR 0.943, CI 95% 0.90-0.98, p = <0.01). Gestational diabetes was associated with development of birth asphyxia (OR 4.00, CI 95% 0.83-19.24, p = <0.06).

Maternal hypertension (p = 0.46), anemia (p = 0.10) and diabetes mellitus (p = 0.93) were not related to an increase risk of birth asphyxia (Table 1).

**Table 1:** **Represents the antepartum risk factors of birth asphyxia in a tertiary care hospital of Karachi, Pakistan 2013**

**Serial no**	**Category**		**Cases N = 123**	**Control N = 117**	**CI/95%**	**P-value**	**OR**
1	**Age of mother**	<20 years	17 (13.8%)	21 (17.9%)	Reference	0.06	3.33
		20-25 years	76 (61.8%)	59 (50.4%)	0.07-1.21	0.09	0.30
		25-30 years	27 (22.0%)	27 (23.1%)	0.15-1.51	0.30	0.14
		30-35 years	3 (2.4%)	10 (8.5%)	0.67-0.95	0.04	0.25
2	**Was mother a booked case?**	Yes	58(37.9%)	95 (62.1%)	0.11-0.37	**<0.01**	0.20
		No	65(74.7%)	22 (25.3%)			
3	**Did the mother suffer from any of these conditions during pregnancy?**						
	a) Maternal hypertension	Yes	14 (11.4%)	10 (8.5%)	0.58-3.52	0.46	1.37
		No	109 (88.6%)	107 (91.5%)			
	b) Gestational diabetes	Yes	8 (6.5%)	2 (1.7%)	0.831	0.06	4.00
		No	115 (93.5%)	115 (98.3%)	19.24		
	c) Anemia	Yes	59 (48%)	44 (37.6%	0.91-2.55	0.10	1.52
		No	64 (52%)	73 (62.4%)			
	d) ante partum hemorrhage	Yes	4 (3.3%)	1 (0.9%)	0.42-35.40	0.19	3.89
		No	119 (96.7%))	116 (99.1%)			
	Pre-eclampsia	Yes	7 (5.7%)	0	0.90-0.98	**<0.01**	0.94
		No	116 (94.3%)	116			
	f) Diabetes mellitus	Yes	5 (4.1%)	5 (4.3%)	0.268-3.367	0.936	0.94
		No	118 (95.9%)	112 (95.7%)			
	g) Placenta pravia	Yes	6 (4.9%)	1 (0.9%)	0.705-50.184	0.064	5.94
		No	117 (95.1%)	116 (99.1%)			

●Threshold of significance is <0.05.

●All significant values were in bold letters.

Intake of Adrenergic drug (OR 0.89 CI 95% 0.84-0.95, p = <0.01) and diuretics (OR 0.91 CI 95% 0.86-0.96, p = <0.01) were associated significantly with increase risk of developing Asphyxia**.**

Infants of primiparous women carried a higher risk for birth asphyxia 70 (56.9%) as compared to normal 39 (33.3%) (OR 2.64 CI 95% 1.56-4.46, p = <0.01). Mother of neonates who developed birth asphyxia was mostly belonging to low socio economic group 62 (50.4%) and they have no prior history of abortion 102 (82.9%). Mothers having prior history of asphyxic child have more prone to birth asphyxia again as compare to control (OR 3.92 CI 95% 1.26-12.19) (Table 2).

**Table 2:** **Represents the antepartum risk factors of birth asphyxia in a tertiary care hospital of Karachi, Pakistan 2013**

**Serial no**	**Category**		**Cases N = 123**	**Control N = 117**	**CI/95%**	**P-value**	**OR**
4	**Was the mother treated with any of the drugs given below**						
	a) Glucocorticoid	Yes	3 (2.4%)	0	0.94-1.00	0.08	0.97
		No	120 (97.6%)	117			
	b) Diuretics	Yes	11 (8.9%)	0	0.86-0.96	**<0.01**	0.91
		No	112 (91.1%)	117			
	c) Antimetabolites	Yes	1 (0.8%)	0	0.97-1.00	0.32	0.99
		No	122 (99.2%)	117			
	d) Ethyl alcohol	Yes	2 (1.6%)	0	0.96-1.00	0.98	0.16
		No	121 (98.4%)	117			
	e) Adrenergic drugs	Yes	13 (10.6%)	0	0.84-0.95	**<0.01**	0.89
		No	110 (89.4%)	117			
5	**Is the mother**	Primigravida	70 (56.9%)	39 (33.3%)	1.56-4.46	**<0.01**	2.64
		Multigravida	53 (43.1%)	78 (66.7%)			
6	**Difference between previous baby and current baby in Multigravida**	1-3 years	40 (93%)	65 (97%)	0.06-2.56	0.32	0.41
		3-6 years	3 (7.0%)	2 (3%)			
7	**Socioeconomic status**	High class	1 (0.8%)	1 (0.9%)	0.07-20.23	0.88	1.24
		High middle class	7 (5.7%)	9 (7.7%)	0.55-4.58	0.38	1.59
		Low middle class	53 (43.1%)	57 (48.7%)	0.78-2.26	0.28	1.33
		Low class	62 (50.4%)	50 (42.7%)	Reference	0.25	1.80
8	**History of abortion**	One abortion	17 (13.8%)	18 (15.4%)	0.67-2.61	0.51	1.27
		Two abortion	4 (3.3%)	11 (9.4%)	1.01-10.74	0.47	3.30
		Three abortion	0	2 (1.7%)	N/A		
		Four abortion	0	1 (0.9%)	N/A		
		No abortion	102 (82.9%)	75 (82.6%)		0.21	0.83

●Threshold of significance is 0.05.

●All significant values were in bold letters.

### Intrapartum risk factors

Although the majority of births take place in hospitals but deliveries that took place at home and private clinics were more prone to the risk of developing Birth asphyxia 27 (22.0%) as compare to control 2 (1.7%) (OR 16.16 CI 95% 3.74-69.75, p = <0.01). Deliveries by midwives were significantly associated with increase risk of birth asphyxia (OR 0.17 CI 95% 0.05-0.51, p = <0.01). Normal vaginal delivery was frequent in both asphyxia 77 (62.6%) and control group 68 (58.1%) and there were also no association to be found with type of anesthesia received by mother. Breech presentation of fetus was significantly associated with increased risk of developing birth asphyxia (OR 2.96 CI 95% 1.25-7.02, p = 0.01) (Table 3).

**Table 3:** **Represents intrapartum risk factors of birth asphyxia in a tertiary care hospital of Karachi, Pakistan 2013**

**Serial no**	**Category**		**Cases N = 123**	**Control N = 117**	**CI/95%**	**P-value**	**OR**
1	**Presentation of fetus**	Cephalic	8 (6.50%)	20 (17.09%)	1.25-7.02	**0.01**	2.96
		Breech	115 (93.5%)	97 (82.905%)			
2	**Mode of delivery**	Normal vaginal delivery	77 (62.6%)	68 (58.1%)	Reference	0.45	0.83
		Delivered by cesarean section before labour (elective)	10 (8.1%)	7 (6.0%)	0.28-2.19	0.65	0.79
		Cesarean section during labour (emergency)	10 (8.1%)	26 (22.2%)	1.32-6.54	<0.01	2.94
		Operated vaginal delivery	26 (21.1%)	16 (13.7%)	0.34-1.46	0.31	0.69
3	**If there was assisted vaginal delivery, was that a**	Ventouse delivery	4 (33.3%)	1 (14.3%)	0.26-34.19	0.36	3.00
			8 (66.7%)	6 (85.7%)			
		Forceps delivery					
4	**If cesarean, anesthesia** received by mother during c-section was	General anesthesia	9 (19.6%)	4 (8.2%)	0.78-9.60	0.10	2.73
		Spinal anesthesia	37 (80.4%)	45 (91.8%)			
5	**Is there any history of prolonged labor?**	Yes	30 (24.4%)	11 (9.4%)	1.47-6.54	**<0.01**	3.09
		No	93 (75.6%)	106 (90.6%)			
6	**Place of delivery**	Home and private clinics hospitals	27 (22.0%)	2 (1.7%)	3.74-69.75	**<0.01**	16.16
			96 (78.0%)	115 (98.3%)			
7	**Delivery conducted by whom?**	Doctor	102 (82.9%)	113 (96.6%)	0.05-0.51	**<0.01**	0.17
		Midwife (dai)	21 (17.1%)	4 (3.4%)			
8	**Referred from**	Emergency	4 (3.3%)	13 (11.1%)	0.58-13.30	0.19	2.78
		Ward	113 (91.9%)	97 (82.9%)	0.23-2.26	0.59	0.79
		Other private and government hospital	6 (4.9%)	7 (6.0%)	Reference	0.78	1.17

●Threshold of significance is 0.05.

●All significant values were in bold letters.

Maternal fever ( OR 10.01 CI 95% 3.78-26.52, p = <0.01), umbilical cord complication like prolapsed umbilical cord (OR 3.36 CI 95% 0.90-12.54, p = 0.05), cephalopelvic disproportion (OR 4.09 CI 95% 1.47-11.53, p = <0.01), prolonged labor (OR 16.16 CI 95% 3.74-69.75, p = <0.01) and Spontaneous premature rupture of membrane (OR 9.25 CI 95% 3.75-22.81, p = <0.01) were significantly associated with increase risk of developing birth asphyxia (Table 4).

**Table 4:** **Represents intrapartum risk factors of birth asphyxia in a tertiary care hospital of Karachi, Pakistan 2013**

**Serial no**	**Category**		**Cases N = 123**	**Control N = 117**	**CI/95%**	**P-value**	**OR**
9	**Did the mother suffer any of the conditions during labor/pregnancy?**						
	a) Prolapsed umbilical cord	Yes	10 (8.1%)	3 (2.6%)	0.90-12.54	**0.05**	3.36
		No	113 (91.9%)	114 (97.4%)			
	b) Cephalopelvic disproportion	Yes	19 (15.4%)	5 (4.3%)	1.47-11.53	<0.01	4.09
		No	104 (84.6%)	112 (95.7%)			
	c) Maternal hypotension	Yes	6 (4.9%)	4 (3.4%)	0.39-5.27	0.57	1.44
		No	117 (95.1%)	113 (96.6%)			
	d) Spontaneous premature rupture of membranes (PROM)	Yes	41 (33.3%)	6 (5.1%)	3.75-22.81		
		No	82 (66.7%)	111(94.9%)		**<0.01**	9.25
	e) Artificial premature rupture of membranes (PROM)	Yes	2 (1.6%)	1 (0.9%)	0.17-21.43		
		No	121 (98.4%)	116 (99.1%)		0.59	1.91
	f) Fever	Yes	38 (30.9%)	5 (4.3%)	3.78-26.52	**<0.0**	10.0
				112		**1**	1
		No	85 (69.1%)	(95.7%)			
	g) Was the mother given any sedative or analgesic drugs intravenously within 1 hour of delivery or intramuscularly within 2 hours of delivery?	Yes	34 (27.9%)	60 (51.3%)	0.21-0.62		
		No	88 (72.1%)	.57 (81.7)		**<0.01**	0.36

●Threshold of significance is 0.05.

●All significant values were in bold letters.

### Fetal risk factors

The risk for developing birth asphyxia was higher in the infant of weight 1–2 kg (OR 0.13 CI 95% 0.05-0.32, p = <0.01) as compared to the infant with weight of 2.5- to >3.5 kg. Prematurity carried a substantially higher risk of developing birth asphyxia, with gestational age of 34 to 37 weeks, increasing the risk of asphyxia by a factor of 0.34 (CI 95% 0.19-0.58). Fetal conditions like oligohydromnios (OR 0.92 CI 95% 0.88-0.97, p = <0.01), pre-mature delivery (OR 26.68 CI 95% 3.54-201.10, p = <0.01) and fetal distress (OR 1.69, CI 95%, 0.00-0.11 p = <0.01) were significantly associated with increment in the risk of developing birth asphyxia. Intra uterine mecoinum release was significantly present in asphyxia cases 24 (19.5%) (p = <0.001). Amniotic fluid surfactant test were found negative only in asphyxia cases 9 (7.37%) (Tables 5 and 6).

**Table 5:** **Represents fetal risk factors of birth asphyxia in a tertiary care hospital of Karachi, Pakistan 2013**

**Serial no**	**Category**		**Cases N = 123**	**Control N = 117**	**CI/95%**	**P-value**	**OR**
1	**Were any fetal conditions (given below) suspected/diagnosed during pregnancy**						
	a) Multiple births	Yes	1 (0.8%)	8 (6.2%)	0.01-0.90	**0.01**	0.11
		No	122 (99.2%)	109 (93.2%)			
	b) Polyhydramnios	Yes	0	2 (1.7%)	0.99-1.04	0.14	1.01
		No	123	115 (98.3%)			
	c) Oligohydramnios	Yes	9 (7.3%)	0	0.88-0.97	**<0.01**	0.92
		No	114 (92.7%)	117			
	d) Meconium-stained amniotic fluid	Yes	24 (19.5%)	0	0.73-0.87	**<0.01**	0.80
		No	99 (80.5%)	117			
	e) Abnormal heart rate or rhythm	Yes	1 (0.8%)	0	0.97-1.00	0.32	0.99
		No	122 (99.2%)	117			
	f) Acidosis (fetal scalp capillary blood)	Yes	4 (3.3%)	0	0.93-0.99	0.96	0.04
		No	119 (97.6%)	117			
	g) Decreased rate of growth (uterine size)	Yes	3 (2.43%)	0	0.94-1.00	0.08	0.97
		No	120 (97.5%)	117			
	h) Premature delivery	Yes	23 (18.7%)	1 (0.9%)	3.54-	**<0.01**	26.68
		No	100 (81.3%)	116 (99.1%)	201.10		
	i) Amniotic fluid surfactant test negative or intermediate within 24 hours of delivery	Yes	9 (7.37%)	0	0.88-0.97	**<0.01**	0.92
		No	114 (92.6%)	117			
	j) Ultrasound guided gross abnormality	Yes	0	1 (0.9%)	0.99-1.02	0.30	1.00
		No	123	116 (99.1%)			
	k) Abnormal striol level	Yes	2 (1.62%)	0	0.96-1.00	0.16	0.98
		No	121 (98.38%)	117			
	L) None	Yes	75 (61.0%)	100 (85.5%)	0.14-0.49	<0.01	0.26
		No	48 (39.0%)	17 (14.5%)			

●Threshold of significance is <0.05.

●All significant values were in bold letters.

**Table 6:** **Represent fetal risk factors of birth asphyxia in a tertiary care hospital of Karachi, Pakistan 2013**

**Serial no**	**Category**		**Cases N = 123**	**Control N = 117**	**CI/95%**	**P-value**	**OR**
2	Was the child resuscitated?	Yes	82 (66.7%)	1 (0.9%) 116	31.27-1720.74	<0.01	232.00
		No	41 (33.3%)	(99.1%)			
3	If yes, then how was the newborn resuscitated	Suction	23 (28.4%)	0	N/A		
		Facial oxygen	28 (34.6%)	1 (100%)	N/A		
		Bag + mask IPPV	20 (24.7%)	0	N/A		
		ET intubation IPPV	6 (7.4%)	0	N/A		
		Medications	4 (4.9%)	0	N/A		
		Vascular resuscitation			N/A		
4	Did the neonate suffer any of the conditions given below?	Cord strangulation around neck	18 (14.63%)	0	N/A		
		Significant fetal distress	37 (30.08%)	2 (1.70%)	0.00-0.11	**<0.01**	0.01
		None	68 (55.28%)	115 (98.29%)	Reference	**<0.01**	1.69
5	Gestational age of the baby at birth	Pre-term	66 (53.7%)	32 (27.4%)	0.19-0.58	**<0.01**	0.34
		Term	54 (43.9%)	77 (65.8%)	reference	**0.04**	1.42
		Post-term	3 (2.4%)	8 (6.8%)	0.47-7.37	0.37	1.87
6	Did the baby cry?	Yes	61 (49.6%)	113 (96.6%)	0.01-0.10	**<0.01**	0.03
		No	62 (51.4%)	4 (3.4%)			
7	Baby weight	1-2 kg	47 (38.2%)	7 (6%)	0.05-0.32	**<0.01**	0.13
		2-2.5 kg	25 (20.3%)	52 (44.4%)	0.94-0.34	**0.05**	1.84
		2.5-3.5 kg	49 (39.8%	57 (48.7%	reference	0.54	1.12
		>3.5 kg	2 (1.6%)	1 (0.9%)	0.28-4.37	0.88	1.10

●Threshold of significance is <0.05.

●All significant values were in bold letters.

## Discussion

According to WHO, 4–9 million newborns develop birth asphyxia each year and at least the same number develop severe consequences such as epilepsy, cerebral palsy and developmental delay [6]. Major manifestations of asphyxia are produced as a result from a combination of hypoxia and ischemia of the brain and other vital organs. It occurs due to combination of vasodilatation and vasoparalysis [6].

Our study objective was to evaluate the Antepartum, Intrapartum and fetal risk factors of Birth asphyxia. In our study Age of mother, lack of booking status, pre-eclampsia, intake of diuretics and adrenergic drugs were reported as maternal risk factors. Significant Intra partum risk factors were home delivery by midwives, breech presentation, prolapsed umbilical cord, cephalopelvic disproportion and fever. Significant Fetal risk factors were oligohydromnios, meconium stained amniotic fluid, pre-mature delivery, resuscitation of neonate pre-term delivery and low birth weight.

Study indicated that young maternal age (20–25 years) and primigravidity has been one of the main risk factors of developing birth asphyxia as mentioned in previous studies [4,11-13]. Pre-term delivery also emerged as one of the significant risk factor of birth asphyxia as reported in past studies [4,12]. It may be due to the fact that pre-term babies face multiple morbidities including organ system, immaturity specially lung immaturation causing respiratory failure [11].

To reduce the risk factors of causing birth asphyxia in low income and developing countries is not an easy task due to certain reasons. One of them was the delivery conducted by untrained traditional midwives as indicated in previous reports also [11,14]. This reflects our limited resources and uneducated rural settings where due to the lack of awareness and resources, home births by untrained midwives were customary [11]. In our settings, mostly deliveries occurred at hospitals. but those births which were take place at home were found to be the significant risk factor for causing birth asphyxia [14]. Findings showed that only less than half of the mothers of affected neonates received counseling regarding birth asphyxia. However majority of women may not be expected to be familiar with or adopt appropriate preventive attitudes with respect to birth asphyxia during their pregnancies as contrary to past studies [13,15]. In order to reduce the burden of birth asphyxia, Women need to educate with not respect to just about her pregnancy but also with respect to the complications which may arise during deliveries.

Two important risk factors mentioned in our study were pre-term delivery and maternal fever, same as mentioned by Lee et al. and Khreisat et al. [11,16]. Their synergy may be explained by a common inflammatory pathway of neonatal brain injury involving cytokines and chemokines which stems from exposure to maternal infection and pre-maturity. Furthermore premature infants are more vulnerable to ischemia due to incomplete blood brain barrier formation [11].

Regarding mode of delivery it showed that most of the cases and control were delivered by normal vaginal delivery, this result was very much similar with the findings of two studies conducted in Pakistan, 2012 on same issue [17,18].

In our study, meconium stained amniotic fluid was found to be present as one of the risk factor, findings were comparable with previous study also [4]. In healthy, well oxygenated fetuses, this diluted meconium is readily cleared from the lungs by normal physiological mechanism, however in few cases meconium aspiration syndrome occurs.

Breech presentation exhibited a 2.96 times higher risk of birth asphyxia than other presentations, results were similar to previous studies [4,19]. It may be due to the fact that breech presentation had higher risk of umbilical cord prolapse, head entrapment, birth trauma and perinatal mortality [4].

Low birth weight was one of the major culprits for causing birth asphyxia [4]. A potential confounder for this could be the fact that mother of low birth weight babies often related to complications such as maternal hypertension and diabetes that present pre-conception or antepartum [10].

Hypertension and anemia were not emerged as a risk factors for birth asphyxia but they found more common in cases as compare to control, same as observed in past studies [8,20]. Hypertension can cause a decrement in blood flow resulting in asphyxia while anemia causes intrapartum hypoxia [8,20].

In our study, socioeconomic factors were associated non-significantly with risk of birth asphyxia same as indicated in past study [21]. Pre-eclampsia found to be associated significantly with increased risk of birth asphyxia [11]. Antepartum hemorrhage, oligohydrominos and fetal distress were reported as a risk factors of birth asphyxia in our study which were contrary to past studies [4,12]. Previous history of birth asphyxia, presence of maternal hypotension, antepartum hemorrhage and diabetes mellitus were not evaluated as a significant risk factors of birth asphyxia but they were more commonly present in mother of effected neonates [4,11,12].

In our study, usage of adrenergic drugs and diuretics were found to be the significant reisk factors of birth asphyxia which was not mentioned in any prior study on this subject.

This study was hospital based and addressed a common problem of our community Majority of study population as not being delivered under care of trained professionals primarily at home and private clinics also reflects another important issue pertaining to mortality and morbidity.

### Strength and limitations

The strength of our study lies in its case control design. Previous studies of Pakistan have targeted few and selected risk factors but we discuss nearly each and every risk factors of birth asphyxia. All attempts were made to ensure that the data collected was reliable and the methods were reproducible. However, our study was not free from limitations.

Main limitation was that our study conducted in one tertiary care hospital of Karachi where mostly patients belong to the low and low middle class and data couldn’t predict the overall situation in the country. Second limitation was short sample size of the study which may have limited our ability to detect small differences. Furthermore, convenient sampling was employed, which may have led to selection bias, and hence was not truly representative of the population under study. Third limitation was that, for the evidence of anemia, diabetes and hypertension we mostly relied on the maternal history without consideration of when the events occurred and how long they were or how they were managed.

### Future researches

Our research opens the door of large proportion of research which needs on this topic to define more accurately the true burden of birth asphyxia in developing countries, contribution of common risk factors such as maternal malnutrition and infection and outcome of asphyxia, and to intervention strategies that can be applied at scale in developing countries.

## Conclusion

Sequelae of birth asphyxia vary from no ill effects to multiorgan complications and death. This diversity varies with severity and duration of asphyxia and it may be due to the presence of gaps in the field of research and technology in developing and low income countries.

Age of mother, lack of booking status, pre-eclampsia, intake of diuretics and adrenergic drugs were reported as maternal risk factors. Intra partum risk factors are home delivery by midwives, breech presentation, prolapsed umbilical cord, cephalopelvic disproportion and fever. Fetal risk factors are oligohydromnios, meconium stained amniotic fluid, pre-mature delivery, resuscitation of neonate pre-term delivery and low birth weight. Majority of these factors may be manageable by means of good pre-natal care.
